# Comparison of direct measures of adiposity with indirect measures for assessing cardiometabolic risk factors in preadolescent girls

**DOI:** 10.1186/s12937-017-0236-7

**Published:** 2017-02-23

**Authors:** Megan Hetherington-Rauth, Jennifer W. Bea, Vinson R. Lee, Robert M. Blew, Janet Funk, Timothy G. Lohman, Scott B. Going

**Affiliations:** 1 Department of Nutritional Sciences, University of Arizona, Tucson, AZ 85721 USA; 2 Departments of Medicine, University of Arizona, Tucson, AZ 85721 USA; 3 University of Arizona Cancer Center, Tucson, AZ 85724 USA; 4 Department of Physiology, University of Arizona, Tucson, AZ 85721 USA; 5 College of Agriculture and Life Sciences, Department of Nutritional Sciences, University of Arizona, Shantz Building, PO Box 210038, 1177 E. 4th Street, Tucson, AZ 85721-0034 USA

**Keywords:** Body composition, Cardiovascular disease, Girls

## Abstract

**Background:**

Childhood overweight and obesity remains high, contributing to cardiometabolic risk factors at younger ages. It is unclear which measures of adiposity serve as the best proxies for identifying children at metabolic risk. This study assessed whether DXA-derived direct measures of adiposity are more strongly related to cardiometabolic risk factors in children than indirect measures.

**Methods:**

Anthropometric and DXA measures of adiposity and a comprehensive assessment of cardiometabolic risk factors were obtained in 288, 9–12 year old girls, most being of Hispanic ethnicity. Multiple regression models for each metabolic parameter were run against each adiposity measure while controlling for maturation and ethnicity. In addition, regression models including both indirect and direct measures were developed to assess whether using direct measures of adiposity could provide a better prediction of the cardiometabolic risk factors beyond that of using indirect measures alone.

**Results:**

Measures of adiposity were significantly correlated with cardiometabolic risk factors (*p* < 0.05) except fasting glucose. After adjusting for maturation and ethnicity, indirect measures of adiposity accounted for 29-34% in HOMA-IR, 10-13% in TG, 14-17% in HDL-C, and 5-8% in LDL-C while direct measures accounted for 29-34% in HOMA-IR, 10-12% in TG, 13-16% in HDL-C, and 5-6% in LDL-C. The addition of direct measures of adiposity to indirect measures added significantly to the variance explained for HOMA-IR (*p* = 0.04).

**Conclusion:**

Anthropometric measures may perform as well as the more precise direct DXA-derived measures of adiposity for assessing most CVD risk factors in preadolescent girls. The use of DXA-derived adiposity measures together with indirect measures may be advantageous for predicting insulin resistance risk.

**Trial registration:**

NCT02654262. Retrospectively registered 11 January 2016.

## Background

With the rising prevalence of childhood obesity and its associated increased risk for developing metabolic abnormalities, type 2 diabetes and cardiovascular disease (CVD), having a measure of adiposity that is accurate and sensitive for predicting CVD risk in children is essential [1]. Currently, the most commonly used index to assess adiposity and identify individuals at cardio-metabolic risk is BMI [2]. Although BMI is an easy index to calculate, it does not differentiate between fat and lean tissue masses [3]. Body fat is more associated with metabolic abnormalities than lean body mass [4]. In children, fat mass and lean mass increase at different proportions depending on pubertal status, making the use of BMI to assess adiposity in children difficult [5]. Indeed, BMI has been shown to have low sensitivity for identifying children at risk for health related problems, with 38% of BMI-defined obese children not having metabolic syndrome (MetS) [6, 7]. This leads to the question as to whether BMI is an adequate measure for assessing adiposity in children and if it should continue to be used as a tool for risk stratification.

Waist circumference (WC) is another common indirect measure of adiposity. Unlike BMI, which is a proxy measure for total body adiposity, WC is an indicator of abdominal adiposity [2]. In adults, it is clear that the central distribution of body fat, particularly visceral adipose tissue, is associated with metabolic impairment and thus is an important measure for predicting risk in adults [6]. However, the relationship between visceral fat and cardiometabolic risk in children may differ from that of adults [8]. In addition, the accuracy of using WC to predict CVD risk in children is complicated by their large variation in growth rates. The height of children of similar age and sex can vary by up to ~28 cm [9]. In adults with the same WC, taller individuals have less metabolic risk than shorter individuals. Hence, the use of waist-to-height ratio (WHtR) for predicting risk in children has been suggested in order to control for the height variablility [9]. Whether WHtR is superior to other adiposity measures for assessing cardiometabolic risk in children is unclear [10].

With the development of imaging methods, total and regional adiposity can now be more accurately and precisely measured. Dual-energy X-ray absorptiometry (DXA) is considered a gold standard for body fat assessment [2]. Unlike the anthropometric measures of adiposity, DXA is able to discriminate between fat mass and lean mass [11]. However, it is costly and involves exposure to radiation making the use of simpler indirect methods of adiposity assessment more feasible for clinical and epidemiological settings. Despite DXA’s ability to accurately measure adiposity, whether it is superior to indirect measures for identifying children with cardiometabolic risk is unclear. Therefore, the aim of this study was to assess whether direct DXA-derived measures of total and regional adiposity are more strongly related to cardiometabolic risk factors in children than common indirect measures.

## Methods

### Study population

Two-hundred eighty-eight girls aged 9–12 were recruited from local schools, pediatric clinics, and wellness community events in Tucson, Arizona as participants in the “Soft Tissue and Bone Development in Young Girls (STAR)” study, designed to assess the effects of adiposity and related metabolic risk factors on bone development. Exclusion criteria included: diagnosis of diabetes, taking any medications that alter body composition, physical disability that limits physical activity, and learning disability that limited completion of questionnaires or otherwise made the participant unable to comply with assessment protocols. The study protocol was approved by the University of Arizona Human Subjects Protection Committee. Written informed consent was obtained from all participants and their parents or legal guardians. Once enrolled, the girls were scheduled to come to the Body Composition Research Laboratory at the University of Arizona where all body composition measurements, blood draw, and questionnaires were completed.

### Anthropometric measures

Anthropometric measures were obtained according to standardized protocols [12]. Body mass was measured to the nearest 0.1 kg using a calibrated scale (Seca, Model 881, Hamburg, Germany) and height was measured at full inhalation to the nearest mm using a stadiometer (Shorr Height Measuring Board, Olney, MD). Using a flexible tape and with the subjects standing, waist circumference (WC) was measured at the umbilicus in cm. The mean of two measurements was used for each anthropometric variable. Measurements were repeated if they differed by ≥0.3 kg for body mass and ≥0.5 cm for height and ≥1 cm for WC. If repeat measures were required, the mean of the second set of measures was used [13]. BMI was calculated as weight (kg) divided by height (m) squared. Based on CDC growth charts, BMI percentiles specific for age and gender were used to categorize girls as either normal weight (≥5th and <85th percentiles), overweight (≥85th and <95th percentiles), or obese (≥95th percentile) [14]. WHtR was calculated as WC (cm) divided by height (cm). Maturity offset was estimated from age and anthropometric measures (height, weight, sitting height, and leg length) using the Mirwald equation [15]. Maturity offset, an estimate of years to peak height velocity, is strongly related to skeletal maturation [15].

### DXA adiposity measures

Measures of whole-body (total fat mass, total percent fat) and regional adiposity (android fat mass) were obtained from dual energy x-ray absorptiometry (DXA) using a GE/Lunar Radiation Corp (Madison, WI) Prodigy following standard subject positioning and data acquisition protocols. The android region is the area extending vertically from immediately superior to the iliac crests to 20% of the distance between this point and immediately below the chin, and laterally to include all of the torso. The within-subject variation for bone and soft tissue in our laboratory on the Lunar Prodigy machine has been previously reported [16, 17]. The DXA was calibrated daily according to manufacturer guidelines. DXA scan analyses were performed by one certified technician. Fat mass index (FMI) was calculated from DXA-derived total body fat mass in kilograms divided by the square of height in meters ((kg)/height^2^ (m)).

### Metabolic measures

Fasting blood samples were drawn by venipuncture and centrifuged after sitting for a minimum of 30 min and no longer than 45 min at room temperature to allow for clotting. Serum was separated and aliquoted into cryovials, and either immediately sent for analysis or stored at -80 °C for later analyses. Fasting glucose and lipids were measured by CLIA certified clinical laboratory immediately post-processing. Fasting glucose was measured in serum utilizing a clinical laboratory hexokinase-based automated assay with intra- and inter-assay variability of 3.22 and 0.54%, respectively. Total fasting triglycerides were measured in serum with an automated enzymatic method using a modified Trinder reaction. The intra- and inter-assay variability were 2.85% and 0.48%, respectively. Total fasting high density lipoprotein cholesterol (HDL-C), and low density lipoprotein cholesterol (LDL-C) were measured in serum with an automated enzymatic assay utilizing a homogeneous method. Intra- and inter-assay variability for HDL-C were 4.12 and 1.25% and for LDL-C were 5.61 and 0.46%. Using stored serum samples, fasting insulin was measured in the laboratory at the University of Arizona utilizing a human insulin specific RIA that does not cross-react with pro-insulin (EMD Millipore, Darmstadt, Germany). The intra- and inter-assay variability were 2.9 and 7.5%, respectively. If any intra-assay difference between duplicates was >10%, the sample was reanalyzed. Insulin resistance was estimated using a homeostatic model assessment of insulin resistance (HOMA- IR), with the following calculation: HOMA-IR = (insulin [μU/L] × glucose [mM/L])/22.5 [18].

### Statistical Analysis

Descriptive statistics were calculated to describe the relevant characteristics of the sample. Descriptive statistics are presented as means ± standard deviations (SD) for normally distributed measures and as median (25th and 75th percentiles) for skewed variables. Bivariate relationships between each indirect adiposity measure and each direct measure were estimated using Pearson’s correlation coefficient and the Spearman rank order correlation coefficient. All correlations were adjusted using a Bonferroni-corrected *p*-value.

Multiple linear regression was used to examine associations of each of the adiposity measurements with cardiometabolic risk factors adjusting for relevant covariates. Six regression models were run for each metabolic parameter. Each model included a single measure of either total or regional body fat to avoid the potential for collinearity as all the body fat measures were strongly inter-correlated. Pearson’s correlations were calculated between potential covariates and each of the metabolic risk indices. Covariates with the highest correlation were included in the regression models in addition to ethnicity, which was included a priori. All models were checked for linearity, normality, and homoscedasticity and appropriate transformations were performed if one or more of these linear regression assumptions were not met. Partial correlations were used to compare the degree of association between the body fat measures and each of the metabolic risk factors after controlling for potential confounders. Ninety-five percent confidence intervals (CI’s) were calculated for each partial correlation coefficient. The squared partial correleation was used to assess the proportion of variance in each metabolic risk factor that was explained by the adiposity measures after adjusting for the other covariates in the regression models. Although we included ethnicity as a covariate in all our models, for a sensitivity analysis, we refit the regression models including only girls who were of Hispanic ethnicity in order to examine if the relationships between the body fat measures and each of the metabolic risk factors differed from the whole study population. Also, for further analysis, we tested whether scaling total percent body fat and android fat by height could increase their individual association with each risk factor.

To assess whether using direct measures of adiposity could provide a better prediction of the cardiometabolic risk factors beyond that of using indirect measures alone, multiple regression models that included all adiposity measures (ie. direct and indirect combined) were developed for each of the cardiometabolic risk factors. A Wald test was performed to test whether the set of direct adiposity measures significantly contributed to the prediction model. For further analysis, a separate regression model for the addition of direct measures of whole body adiposity (FMI, total % fat) to an indirect whole body measure (BMI) were performed against each metabolic factor to assess if the addition of whole body direct measures to whole body indirect measures of adiposity added significantly to the overall variance in the metabolic risk factors beyond that explained by indirectly assessed whole body adiposity. Similar regression models were made for the addition of the direct measure of regional adiposity (android fat mass) to indirect regional measures (WC, WHtR).

To assess if the amount of variance in each of the metabolic risk factors explained by the combination of whole body adiposity measures differed when using indirect vs. direct measurement methods, the adjusted R^2^ from regression models with an indirect whole body adiposity measure (BMI) were compared to the adjusted R^2^ of models with a direct measure of whole body adiposity (total % fat). Similarly, to assess if the amount of variance in each of the metabolic risk factors explained by the combination of regional adiposity measures differed when using indirect vs. direct measurement methods, the adjusted R^2^ from regression models with an indirect regional adiposity measure (WC or WHtR) were compared to the adjusted R^2^ of models with an indirect measure of regional adiposity (android fat mass). Partial correlation coefficients of the regional and whole body measures in the previously described models were compared to assess whether whole body measures of adiposity had greater associations with the metabolic risk factors than regional measures. As there was a high potential for collinearity, the variance inflation factors (VIF) were calculated for all models including multiple adiposity measures.

A *p*-value of <0.05 was considered statistically significant. All analyses were performed using STATA version 13.1.

## Results

Of the 288 girls recruited for the study, 269 girls had complete data for all variables and were used in the final analyses except for regression models with HOMA-IR for which 234 girls made up the total sample due to study budgetary constraints for measuring fasting insulin levels. Similar results were found when regression analyses were run on other risk factors based on 269 or 234 girls. Sample descriptive statistics are given in Table 1. All variables are presented in their original units. The cardiometabolic parameters (except for fasting glucose), WC, WHtR, FMI and Android fat, were not normally distributed and were log-transformed for linear regression analyses. The majority (76%) of the sample reported Hispanic ethnicity. Based on U.S. National Center for Health Statistics/Centers for Disease Control and Prevention percentiles for body mass index (BMI, kg/m^2^), 59% of girls were normal weight (BMI 5th–85th percentile), 18% were overweight (BMI 85th–95th percentile), and 23% were obese (BMI >95th percentile) [19].

**Table 1:** Participant characteristics

Characteristic	Mean ± SD
Age (years)	10.8 ± 1.1
Ethnicity	
Hispanic or Latino	205 (76.2%)
Not Hispanic or Latino	64 (23.8%)
Weight (kg)	41.9 (33.6–53.2)^b^
Height (cm)	146.0 ± 9.6
BMI Percentile	
Normal (<85th)	159 (59.1%)
Overweight (≥85th < 95th)	47 (17.5%)
Obese (≥95th)	63 (23.4%)
WC (cm)	73.3 (64.1–84.0)^b^
WHtR	0.5 (0.4–0.6)^b^
Body fat, %	32.4 ± 10.0
FMI (kg/m^2^)	6.1 (3.9–9.2)^b^
Android fat mass (kg)	0.9 (0.5–1.7)^b^
Fasting Glucose (mg/dL)	92.9 ± 6.8
Fasting Insulin (μU/mL)^a^	17.8 (13.3–23.9)^b^
HOMA-IR^a^	4.0 (3.0–5.6)^b^
TG (mg/dL)	90.0 (69–126)^b^
HDL (mg/dL)	50.0 (45–58)^b^
LDL (mg/dL)	97.0 (83–119)^b^

*WC* waist circumference (cm), *WHtR* waist-to-height-ratio, *FMI* fat mass index (kg/m^2^), *HOMA*-*IR* homeostatic model assessment of insulin resistance, *TG* triglycerides, *HDL*-*C* high density lipoprotein cholesterol (mg/dL), *LDL*-*C* low density cholesterol (mg/dL)

^a^ *n* = 234

^b^median (range- 25th percentile to 75th percentile)

Pairwise correlation coefficients between the indirect and direct measures of body fat are given in Table 2. All adiposity measures were strongly inter-correlated. BMI-percentile, BMI category (normal weight, overweight, and obese), and BMI were most strongly correlated with FMI. WC was most strongly correlated with DXA Android fat mass whereas WHtR had a stronger correlation with DXA total body % fat and FMI, both of which are measures of total adiposity rather than regional adiposity.

**Table 2:** Pairwise correlation coefficients among direct and indirect adiposity measures

Indirect adiposity measure	Direct adiposity measure		
	Total % body fat	logFMI	logAndroid fat mass
BMI percentile	0.85	0.90	0.88
BMI	0.87	0.92	0.90
BMI category^a^	0.84	0.87	0.85
logWC	0.89	0.92	0.94
logWHtR	0.89	0.90	0.87

*WC* waist circumference (cm), *WHtR* waist-to-height-ratio

^a^Spearman correlation used

*n* = 269

*p* > 0.005 for all correlations

Results of linear regression between each adiposity measure with the cardiometabolic risk factors are presented in Table 3. All models were adjusted for ethnicity and maturity offset, a measure of skeletal maturation. Adjustment for other potential confounding factors such as physical activity and diet did not substantially alter partial correlations between adiposity measures and metabolic risk factors (<0.03 change in r) and added <1% to the total variance explained by the models (data not shown). Thus, they were not included in the final models. In addition, BMI categories based on established cut points [19] were used in all regression analyses as these categories explained more of the variance in the metabolic risk factors than BMI as a continuous variable (data not shown). Overall, after adjusting for ethnicity and maturation, the variances in cardiometabolic indices explained by indirect and direct adiposity measures were similar and their partial correlations fell within 95% CI’s of each other, with the exception of the overweight BMI category, which had weaker association with each of the metabolic factors (*r* = 0.32, 0.31, 0.14, -0.13, 0.09 for fasting insulin, HOMA-IR, TG, HDL-C, and LDL-C respectively). All other indirect measures of adiposity accounted for 30-36% of the variance in fasting insulin, 0.4-0.6% in fasting glucose, 29-34% in HOMA-IR, 10-13% in TG, 14-17% in HDL-C, and 5-8% in LDL-C while direct measures accounted for 30-36% in fasting insulin,﻿﻿ ﻿0.6-10% in fasting glucose, 29-34% in HOMA-IR, 10-12% in TG, 13-16% in HDL-C, and 5-6% in LDL-C. Of the indirect measures, BMI categories had weaker associations with each of the metabolic risk indices compared to WC and WHtR. Compared to the normal weight category, the obese BMI category had stronger associations with each of the metabolic indices, except fasting glucose, than the overweight group (obese vs. overweight group partial correlation for fasting insulin = 0.55 vs. 0.32, fasting glucose = 0.06 vs. 0.09, HOMA-IR = 0.54 vs. 0.31, TG = 0.28 vs. 0.14, HDL-C = -0.38 vs. -0.14, LDL-C = 0.22 vs. 0.09). Both WC and WHtR predicted each metabolic marker to a similar extent (WC vs. WHtR partial correlation for fasting insulin = 0.60 vs 0.57, fasting glucose = 0.08 vs. 0.08; HOMA-IR = 0.58 vs. 0.55, TG = 0.36 vs. 0.35, HDL-C = -0.41 vs. -0.40, LDL-C = 0.28 vs. 0.29). Of the direct measures, Android fat mass explained more of the variance for the majority of metabolic markers compared to total % body fat and FMI after adjusting for maturation and ethnicity (Table 3). None of the adiposity measures were significantly (*p* < 0.05) associated with fasting glucose. The variances in fasting insulin accounted for by both direct and indirect adiposity measures were similar to that of HOMA-IR (Table 3). Since HOMA-IR is based on fasting glucose and insulin, it appears that insulin levels drive the association of HOMA-IR with adiposity.

**Table 3:** Partial correletions of adiposity measures with metabolic risk factors

Metabolic risk marker as dependent variable	Adiposity measure as independent variable	Adiposity Measure Partial correlation (r)	Partial Correlation 95% CI	Model Adjusted R^2^
logFasting Insulin (μU/mL)^a^	*Indirect Measures*			
	BMI category			0.42
	Overweight	0.32***	(0.20, 0.43)	
	Obese	0.55***	(0.45, 0.63)	
	logWC (cm)	0.60***	(0.51, 0.68)	0.45
	logWHtR	0.57***	(0.48, 0.65)	0.42
	*Direct Measures*			
	Total % body fat	0.55***	(0.45, 0.63)	0.41
	logFMI (kg/m^2^)	0.57***	(0.48, 0.65)	0.42
	logAndroid fat mass (kg)	0.60***	(0.51, 0.68)	0.45
logFasting Glucose (mg/dL)^b^	*Indirect Measures*			
	BMI category			0.06
	Overweight	0.09	(−0.03, 0.21)	
	Obese	0.06	(−0.06, 0.18)	
	logWC (cm)	0.08	(−0.04, 0.20)	0.06
	logWHtR	0.08	(−0.04, 0.20)	0.06
	*Direct Measures*			
	Total % body fat	0.09	(−0.03, 0.21)	0.06
	logFMI (kg/m^2^)	0.08	(−0.04, 0.20)	0.06
	logAndroid fat mass (kg)	0.10	(−0.02, 0.22)	0.07
logHOMA-IR^b^	*Indirect Measures*			
	BMI category			0.41
	Overweight	0.31***	(0.19, 0.42)	
	Obese	0.54***	(0.44, 0.63)	
	logWC (cm)	0.58***	(0.49, 0.66)	0.44
	logWHtR	0.55***	(0.45, 0.63)	0.42
	*Direct Measures*			
	Total % body fat	0.54***	(0.44, 0.63)	0.41
	logFMI (kg/m^2^)	0.55***	(0.45, 0.63)	0.42
	logAndroid fat mass (kg)	0.58***	(0.49, 0.66)	0.45
logTG (mg/dL)^b^	*Indirect Measures*			
	BMI category			0.12
	Overweight	0.14*	(0.02, 0.26)	
	Obese	0.28***	(0.17, 0.39)	
	logWC (cm)	0.36 ***	(0.25, 0.46)	0.17
	logWHtR	0.35 ***	(0.24, 0.45)	0.16
	*Direct Measures*			
	Total % body fat	0.28***	(0.17, 0.39)	0.12
	logFMI (kg/m^2^)	0.31***	(0.20, 0.41)	0.13
	logAndroid fat mass (kg)	0.34***	(0.23, 0.44)	0.15
logHDL (mg/dL)^b^	*Indirect Measures*			
	BMI category			0.17
	Overweight	−0.14*	(−0.25,−0.02)	
	Obese	−0.38***	(−0.48,−0.27)	
	logWC (cm)	−0.41***	(−0.51,−0.30)	0.20
	logWHtR	−0.40 ***	(−0.50,−0.29)	0.19
	*Direct Measures*			
	Total % body fat	−0.36***	(−0.46,−0.25)	0.16
	logFMI (kg/m^2^)	−0.39***	(−0.49,−0.28)	0.18
	logAndroid fat mass (kg)	−0.40***	(−0.50,−0.29)	0.19
logLDL (mg/dL)^b^	*Indirect Measures*			
	BMI category			0.04
	Overweight	0.09	(−0.03, 0.21)	
	Obese	0.22**	(0.10, 0.33)	
	logWC (cm)	0.28***	(0.17, 0.39)	0.07
	logWHtR	0.29***	(0.18, 0.40)	0.08
	*Direct Measures*			
	Total % body fat	0.23**	(0.11, 0.34)	0.05
	logFMI (kg/m^2^)	0.25***	(0.13, 0.36)	0.05
	logAndroid fat mass (kg)	0.23***	(0.11, 0.34)	0.05

All models adjusted for ethnicity and maturity offset

*WC* waist circumference (cm), *WHtR* waist-to-height-ratio, *FMI* fat mass index (kg/m^2^), *HOMA*-*IR* homeostatic model assessment of insulin resistance, *TG* triglycerides, *HDL*-*C* high density lipoprotein cholesterol (mg/dL), *LDL*-*C* low density cholesterol (mg/dL)

^a^ *n* = 234

^b^ *n* = 269

*p < 0.05

**p < 0.001

***p < 0.0001

Scaling the adiposity measures of total % body fat and Android fat mass by height, did not increase their ability to predict the metabolic risk factors (data not shown) and thus, total % body fat and Android fat mass were used in all models. When examining the relationship between each adiposity measure and metabolic risk factor in only girls of Hispanic ethnicity, similar partial correlations and model adjusted R^2^’s were found as in the full study sample (data not shown). Thus, to maximize statistical power, results from the full sample are presented in all the tables.

To determine whether direct measures of adiposity added to the variance explained for each metabolic risk marker beyond that explained by indirect measures, the adjusted R^2^ from models including all indirect adiposity measures were compared to models where direct measures were added (Table 4). The addition of both whole body and regional directly assessed adiposity measures (total % body fat, FMI, Android fat mass) to indirect measures (BMI, WC, WHtR) did not significantly increase the variance in TG, HDL-C, and LDL-C explained by indirect measures alone (*p* = 0.05, 0.25, and 0.50, respectively). The addition of direct measures to indirect did add significantly to the variance explained for HOMA-IR (*p* = 0.04). When comparing the addition of direct measures of whole body adiposity (total % body fat, FMI) to the indirect measure of whole body adiposity (BMI), the addition of the direct measures significantly added to the variance explained for all metabolic indices (*p* = 0.002, *p* = 0.03, *p* = 0.02, *p* = 0.03 for HOMAIR, TG, HDL-C, LDL-C respectively). When comparing the addition of DXA-derived android fat mass, a more precise measure of regional fat to the indirect measures of regional adiposity (WC, WHtR), the addition of Android fat mass added significantly to the variance explained for HOMA-IR (*p* = 0.01) but not to TG, HDL-C and LDL-C (*p* = 0.85, 0.21, and 0.20, respectively).

**Table 4:** Variance (adjusted R^2^) in metabolic risk markers explained by all indirect adiposity measures compared to all indirect and direct adiposity measures combined

Measures of Total and Regional Adiposity	Indirect : BMI category, logWC, logWHtR	Indirect + Direct: BMI category, logWC, logWHtR + total% body fat, logFMI, logAndroid fat mass	Significance of adding one or more direct measures
logHOMA-IR	0.45	0.46	*P* = 0.04
logTG	0.14	0.16	*P* = 0.05
logHDL	0.18	0.18	*P* = 0.25
logLDL	0.09	0.08	*P* = 0.50
Measures of Total body adiposity	Indirect: BMI category	Indirect + Direct: BMI category + total % body fat, logFMI	
logHOMA-IR	0.41	0.44	*P* = 0.002
logTG	0.09	0.11	*P* = 0.03
logHDL	0.15	0.18	*P* = 0.02
logLDL	0.04	0.06	*P* = 0.03
Measures of Regional adiposity	Indirect: logWC, logWHtR	Indirect + Direct: logWC, logWHtR + logAndroid fat mass	
logHOMA-IR	0.44	0.46	*P* = 0.01
logTG	0.14	0.14	*P* = 0.85
logHDL	0.17	0.18	*P* = 0.21
logLDL	0.08	0.09	*P* = 0.20

*WC* waist circumference (cm), *WHtR* waist-to-height-ratio, *FMI* fat mass index (kg/m^2^), *HOMA*-*IR* homeostatic model assessment of insulin resistance, *TG* triglycerides, *HDL*-*C* high density lipoprotein cholesterol (mg/dL), *LDL*-*C* low density cholesterol (mg/dL)

All models adjusted for ethnicity and maturity offset

*n* = 234 for all models

When WC or WHtR were added to a regression model with the BMI categories along with ethnicity and maturation, they explained similar amounts of variance in each metabolic factor as the combination of direct measures of total percent fat and android fat mass (Tables 5 and 6). Further comparison of the partial regression coefficients of the BMI categories and WC or WHtR from the models showed that WC and WHtR had higher associations with the metabolic risk factors than the BMI categories (Table 5). Due to the presence of collinearity (VIF > 10) when total percent fat and android fat mass were combined in the same model, we were unable to report meaningful partial correlation coefficients for these measures.

**Table 5:** Partial correlation coefficients and adjusted R^2^ values for regression of BMI categories with either the addition of logWC or logWHtR with metabolic risk factors

BMI category + logWC					BMI category + logWHtR			
	n	Partial Correlation, BMI category	Partial Correlation, logWC	Model Adjusted R^2^	n	Partial Correlation, BMI category	Partial Correlation, logWHtR	Model Adjusted R^2^
logHOMA-IR	234	Overweight:	0.26***	0.45	234	Overweight:	0.20*	0.43
		0.10				0.13*		
		Obese:				Obese:		
		0.14*				0.19*		
logTG	269	Overweight:	0.23***	0.16	269	Overweight:	0.22**	0.16
		−0.04				−0.02		
		Obese:				Obese		
		−0.03				−0.02		
logHDL	269	Overweight:	−0.19*	0.20	269	Overweight:	−0.19*	0.20
		0.01				0.02		
		Obese:				Obese:		
		−0.07				−0.08		
logLDL	269	Overweight:	0.18*	0.07	269	Overweight:	0.20**	0.07
		−0.05				−0.06		
		Obese:				Obese:		
		−0.03				−0.04		

All models adjusted for ethnicity and maturity offset

*WC* waist circumference (cm), *WHtR* waist-to-height-ratio, *FMI* fat mass index (kg/m^2^), *HOMA*-*IR* homeostatic model assessment of insulin resistance, *TG* triglycerides, *HDL*-*C* high density lipoprotein cholesterol (mg/dL), *LDL*-*C* low density cholesterol (mg/dL)

*p < 0.05

**p < 0.001

***p < 0.0001

**Table 6:** Adjusted R^2^ values for regression of total percent body fat and logAndroid fat mass on metabolic risk factors

%total body fat + logAndroid fat mass		
	n	Model Adjusted R^2^
logHOMA-IR	234	0.45
logTG	269	0.17
logHDL	269	0.19
logLDL	269	0.04

All models adjusted for ethnicity and maturity offset

*HOMA*-*IR* homeostatic model assessment of insulin resistance, *TG* triglycerides, *HDL*-*C* high density lipoprotein cholesterol (mg/dL), *LDL*-*C* low density cholesterol (mg/dL)

## Discussion

The aim of this study was to determine whether direct DXA-derived measures of total and regional adiposity are more strongly related to cardiometabolic risk factors in children than common indirect measures. Our results showed that after adjusting for maturation and ethnicity direct and indirect measures of adiposity explained similar amounts of variance in each of the cardiometabolic risk factors with the variance explained being highest for HOMA-IR and fasting insulin and relatively low for TG, HDL-C, LDL-C, and fasting glucose. Considering that the adiposity measures, both direct and indirect, were substantially intercorrelated, the similarities in variance in each metabolic risk factor explained by the measures of adiposity are not surprising. These results suggest there is little to gain by having direct measures of adiposity when it comes to assessing cardiometabolic risk. Similar results were reported by Steinberger et al. who compared BMI and skinfolds to DXA-derived measures of adiposity and the relation of these measures to cardiovascular risk in adolescent boys and girls [20]. That study found that both the indirect anthropometric measures and DXA adiposity measures correlated similarly and to the same magnitude to TG, HDL-C, fasting insulin, and glucose utilization with the strongest relationship occurring in the later 2 metabolic risk indices [20]. Thus, although both indirect and direct adiposity measures can equally predict metabolic risk factors, the degree of relationship differs depending on the specific risk factor being assessed. For instance, the highest amount of variance explained by both the indirect and direct adiposity measures were for fasting insulin and HOMA-IR. This is consistent with previous research, which suggests a strong link between adiposity, insulin signaling, and the development of insulin resistance [21]. Even though HOMA-IR is based on both fasting insulin and glucose (insulin [μU/L] × glucose [mM/L])/22.5), it appears that the association of adiposity with HOMA-IR is mostly driven by fasting insulin since there were no significant correlations between the adiposity measures and fasting glucose levels in our sample where the majority had glucose values within the normal range. This is not surprising since fasting glucose is maintained within a narrow range with little variation whereas there can be up to a 53-fold variation in fasting insulin values in children [22]. This leaves values of insulin to outweigh values of glucose and be the major determinant of their combined effect in HOMA-IR [22, 23]. Overall, our results indicate that a high percentage of variance in insulin levels and in turn HOMA-IR can be explained by changes in adiposity. Besides adiposity, there are other factors that could be influencing levels of the metabolic markers. For instance, it has been shown that LDL-C has a large genetic component contributing to its variation as is also true of HDL-C, with heritability estimates of 40–60% [24]. This might partially explain why we found the indirect and direct adiposity markers to explain lower amounts of variance in TG, HDL-C, and LDL-C within our sample of girls.

When assessing a combination of indirect measures (BMI, WC, and WHtR) instead of each measure individually, we found that the combination of indirect measures explained a high proportion of the variance in the metabolic risk factors and that the addition of direct measures did not increase the variance explained for TG, HDL-C, and LDL-C levels beyond that of the combined indirect measures. However, having more precise DXA-measurements of adiposity did increase the amount of variance explained for HOMA-IR beyond that of what could be explained with just the combination of indirect measures. It is possible that body fat more directly influences changes in insulin sensitivity, whereas high triglyceride and cholesterol levels are a byproduct of insulin resistance. Previous studies have demonstrated in selected subgroups that it is insulin resistance and not the excess adiposity per se associated with obesity that is connected with lower HDL-C and higher triglyceride [25]. Insulin resistance at the adipocyte results in increased release of free fatty acids which in turn stimulates the assembly and secretion of VLDL from the liver resulting in hypertriglyceridemia leading to low HDL-C and increased small dense LDL particles [26]. Hence, fat appears to be a direct factor contributing to insulin resistance, whereas adiposity effects circulating levels of lipids, particularly TG and HDL-C, indirectly via it’s influence on insulin resistance.

The utility of BMI for assessing adiposity and predicting cardiovascular risk factors in children has been questioned as this age group is growing and changes in BMI can reflect increases in lean mass more than fat mass [27]. In the current study we found that relative to normal weight, the BMI overweight category explained the least amount of variance in each of the metabolic risk factors among all the indirect and direct adiposity measures we examined compared to the obese group. Thus, the obese category appears to be the main contributor to the relationship between BMI and metabolic risk factors. Ado et al. also found that BMI in general performed weaker at estimating HOMA-IR, TG, and total cholesterol relative to DXA-measured total percent fat in U.S. children aged 12–18 [28]. When we added the DXA-derived whole body measures of total percent body fat and FMI to BMI, the variance explained for HOMA-IR, TG, HDL-C, and LDL-C levels significantly increased. Likewise, Lawlor et al. found that adding other adiposity measurements to a model with BMI increased the ability to predict risk compared with BMI alone [27].

WC and WHtR have been proposed as being useful indicators of metabolic risk since these measures potentially reflect the regional distribution of fat in the abdomen, which in adults has been shown to relate to abnormal cardiometabolic risk factors more strongly than measures of general adiposity [29]. In our study, we found that both WC and WHtR explained more variance in each of the metabolic risk factors than BMI categories and were comparable in magnitude to the DXA-derived measures of regional and whole body fat. This is contrary to the findings of Freedman et al. who reported that BMI related similarly to CVD risk factors as other indirect and direct measures of body fat in US children [30]. We also found that when WC or WHtR were combined with BMI, both WC and WHtR accounted for greater proportions of the variance in the metabolic risk factors than the BMI categories. These findings are similar to those of Wolfgram et al. who found that WC correlated more strongly than BMI Z-score and to a similar magnitude as MRI-derived adiposity measures with HOMA-IR, TG, and HDL-C in non-obese female adolescent girls of varying ethnicities [31]. Overall, we conclude that indirect regional adiposity measures of WC and WHtR are strongly related to cardiometabolic risk factors in preadolescent girls with no superiority of WHtR over WC. In addition there appears to be no added benefit of having a more direct measure of abdominal adiposity for explaining the variance of metabolic risk indices.

The present study has several strengths. First, we had a relatively large sample of preadolescent girls within a narrow age range and wide range of body fat levels, which allowed for the relationships between fat measures and metabolic risk factors to be determined for a range of body fat without the added confounding effects of gender and age. In addition, we were able to better examine the relationship between the adiposity measures and cardiometabolic factors by controlling for other variables potentially influencing this relationship such as maturation, ethnicity, physical activity, and diet, although in our analyses we found physical activity and diet did not significantly influence the associations between adiposity measures and metabolic risk. Our study adds to the literature examining the association of indirect vs. direct measures of adiposity to cardiometabolic risk as our sample was approximately 80% Hispanic. Few previous studies have focused on Hispanic children, who have been reported to have higher rates of obesity, metabolic syndrome, and diabetes than their non-Hispanic peers [2, 32]. In a sub-analysis of data on Mexican American children aged 12–19 from the National Health and Nutrition Examination Survey 1999–2004, Cui et al. found that whole body adiposity measures, BMI, total percent fat, and FMI were similarly correlated with cardiovascular risk factors with the exception of fasting insulin for which BMI had a stronger relationship [11]. The regional adiposity measures of WC, WHtR, and DXA percent trunk fat, did not significantly differ in their relationship with metabolic risk factors [11]. Our study extends Cui’s findings to Hispanic girls of younger age showing that anthropometric methods were comparable to DXA adiposity measures when assessing metabolic risk. We found no advantage of BMI over DXA measures for assessing fasting insulin, which could indicate that certain measures of adiposity may be better at accessing certain metabolic indices in different age groups.

Since the data for this analysis were cross-sectional, we could not address whether direct measures of adiposity have added value over indirect in determining metabolic risk factors as children progress through puberty and into adulthood, a time when fat patterning and distribution undergo large changes [33]. Future studies are needed to examine if indirect measures of adiposity are adequate enough to capture these changes in adiposity depots, which may reflect changes in metabolic risk factors. In addition, the lipid levels of our study sample were essentially normal, with >80% of the girls falling below the NCEP recommended cut-off for high lipid levels in children [34]. Future research is needed to assess whether direct measures may be advantageous beyond using indirect measures in children with dyslipidemia. A possible limitation of our analysis is the categorization of BMI into normal weight, overweight, and obese groups instead of using BMI as a continuous predictor in our regression models. Such categorization of continuous predictors in linear regression can lead to loss of information and power [35]. However, we found that the categorization of BMI based on established cut points [19] explained more of the variance in the metabolic risk factors than BMI as a continuous variable.

## Conclusions

When it comes to assessing metabolic risk in preadolescent girls, anthropometric measures appear to perform just as well as the more precise direct DXA-derived measures. Out of the anthropometric adiposity measures, WC and WHtR, which perform similarly, are superior to the use of BMI categories for explaining variance in metabolic risk factors. When used in combination there is little additional gain of having direct measures over these indirect measures for identifying metabolic risk factors. However, the use of DXA-derived adiposity measures with indirect measures may be advantageous in the assessment of insulin resistance. Although anthropometric dimensions do not give a precise quantification of whole body or regional fat, these surrogate measures are no less associated with cardiometabolic outcomes than direct measures and thus can be reliably used for determining adverse metabolic risk profiles.

## Abbreviations

BMI: Body mass index
CVD: Cardiovascular disease
DXA: Dual-energy X-ray absorptiometry
FMI: Fat mass index
HDL-C: High density lipoprotein cholesterol
HOMA- IR: Homeostatic model assessment of insulin resistance
LDL-C: Low density lipoprotein cholesterol
MetS: Metabolic syndrome
WC: Waist circumference
WHtR: Waist-to-height ratio
